# Refusal to ICU admission based on futility

**DOI:** 10.1186/2197-425X-3-S1-A474

**Published:** 2015-10-01

**Authors:** O Moreno, FM Acosta, M Muñoz, AM Perez, ME Poyatos, M Colmenero

**Affiliations:** Hospital Universitario San Cecilio, ICU, Granada, Spain

## Objectives

To describe the epidemiology, frequency, criteria and implications of the refusal to ICU admission based on futility in an ICU of 18 beds, in Granada, Spain.

## Methods

Observational study in the time interval from July 2013 to December 2014 (18 months). We prospectively registered all patients refused to admission in our unit, then we analyze the clinical refusal report used in our hospital. From these reports we extracted several variables: demographical (age, sex), origin (emergency room, hospital), clinical (comorbidity), functional situation (based on Stein and Langfitt modified scale), diagnosis, reason of refusal (“too good”, “too bad”, futility, lack of beds, patient refusal), if it was definitive or conditional, if the patient was admitted in ICU afterwards (in the same episode of previous request), and the status at hospital discharge. We realized a descriptive (by frequencies) and multivariable analysis of the factors related to futility refusal.

## Results

There were a total of 547 refusals, which represents 25% of total ICU patients evaluated for admission. From all these, we discarded the cardiologic patients who were rejected because they were “too good” (173), most of them evaluated for thoracic pain.

From the remaining 374, 195 (52.14%) were female, the mean age was 69 ± 7 years (19-98). 185 patients (49.3%) had more than two comorbidities (multipathological) and 183 (48.9%) moderate to severe functional disability. The causes of refusal were: 176 (47.1%) the patient was “too good”, 167 (44.7%) related to qualitative futility, 24 (6.4%) was “too bad”, 4 (1.1%) a lack of beds and in 3 cases (1%) because of patient refusal.

**Figure 1:**
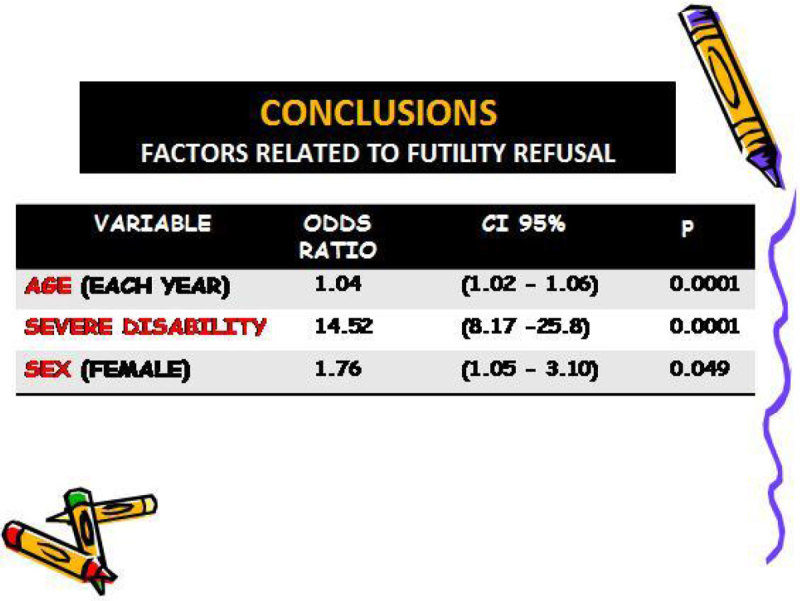
Figure 1

In the multivariable analysis (Figure 1) the significant variables related to futility refusal were age (by years) with an OR of 1.04, CI 95(1.02-1.06) and p < 0.001, severe functional disability with an OR of 14.52, CI 95(8.17-25.8) and p < 0.001, and the sex (female) with an OR of 1.76, CI 95(1.05-3.1) and p = 0.049.

## Conclusions

The refusal to ICU admission is a frequent medical activity in our daily job. The type of patient most rejected is the cardiologic, mostly evaluated for thoracic pain (probably ischemic with low risk). In patients in whom we decided rejection based on subjective qualitative futility, this was mostly related to age, prior functional disability and sex (female).

